# Minimally Invasive Hepatic Resection Option for Access to the Waiting List of a Single Regional Transplant Center in Southern Italy: Entry and Dropout Flows’ Analysis

**DOI:** 10.3390/jcm14248871

**Published:** 2025-12-15

**Authors:** Roberta Vella, Duilio Pagano, Fabrizio di Francesco, Sergio Li Petri, Pasquale Bonsignore, Noemi Di Lorenzo, Sergio Calamia, Alessandro Tropea, Irene Vitale, Ivan Vella, Caterina Accardo, Sandro Gelsomino, Salvatore Vieni, Calogero Cammà, Giovanni Ferrandelli, Salvatore Gruttadauria

**Affiliations:** 1 Abdominal Center, IRCCS-ISMETT, UPMC, 90127 Palermo, Italy; roberta.vella02@unipa.it (R.V.); dpagano@ismett.edu (D.P.); fdifrancesco@ismett.edu (F.d.F.); slipetri@ismett.edu (S.L.P.); pbonsignore@ismett.edu (P.B.); noemidilorenzo96@gmail.com (N.D.L.); scalamia@ismett.edu (S.C.); atropea@ismett.edu (A.T.); ivitale@ismett.edu (I.V.); ivella@ismett.edu (I.V.); caccardo@ismett.edu (C.A.); sgelsomino@ismett.edu (S.G.); gferrandelli@ismett.edu (G.F.); 2 Department of Precision Medicine in the Medical, Surgical and Critical Care Area, University of Palermo, 90133 Palermo, Italy; salvatore.vieni@unipa.it; 3 Gastroenterology and Hepatology Unit, Department of Health Promotion, Mother and Child Care, Internal Medicine and Medical Specialties, Section of Legal Medicine, University of Palermo, 90133 Palermo, Italy; calogero.camma@unipa.it; 4 Department of General Surgery and Medical-Surgical Specialties, University of Catania, 95124 Catania, Italy

**Keywords:** laparoscopic liver resection, waiting list, drop out, liver transplantation, hepatic resection

## Abstract

**Background/Objectives**: The increasing adoption of laparoscopic liver resection (LLR) and changes in clinical management may influence access to curative treatments for patients on the liver transplant waiting list. We aimed to analyze temporal trends in LLR use and to explore the association between the proportion of LLR and the dropout rate from the intention-to-treat (ITT) population. A secondary objective was to assess the risk of dropout or death versus curative treatment (transplantation or resection) in patients with hepatocellular carcinoma (HCC) compared with non-HCC candidates using a competing-risk model. **Methods**: We performed a retrospective cohort study of all patients listed for liver transplantation between 2015 and 2023. Annual rates of LLR and dropout were calculated, and their correlation was evaluated using Spearman’s rho. The risk of dropout/death and competing curative events (OLT, resection, or thermal ablation) was assessed using Fine–Gray competing-risk regression, adjusted for HCC status. **Results**: From 2015 to 2023, LLR accounted for a progressively increasing proportion of liver resections. A significant negative correlation was observed between annual LLR rates and dropout rates (ρ = −0.78, *p* = 0.008), indicating fewer ITT failures with greater LLR adoption. In the competing-risk analysis, HCC patients had a significantly lower subdistribution hazard for dropout/death (SHR 0.27, 95% CI 0.18–0.42, *p* < 0.001) and a higher probability of receiving a curative treatment (SHR 1.65, 95% CI 1.40–1.94, *p* < 0.001). **Conclusions**: The increased use of LLR was associated with improved access to curative therapies and a reduced dropout risk on the liver transplant waiting list. HCC patients showed a more favorable competing-risk profile compared with non-HCC candidates.

## 1. Introduction

Hepatocellular carcinoma (HCC) is the most common primary liver tumor [1] and both liver resection and transplantation are considered potentially curative options. Liver transplantation is recognized as the most radical oncological treatment for HCC, with a 5-year overall survival and disease-free survival of 70% and 90%, respectively [1]. However, the shortage of available organs represents a critical issue since it prolongs the waiting list time that is associated with tumor progression and deteriorating liver function, resulting in an increased risk of drop-out. Low donation rate in southern Italy and constant influx of new patients with a diagnosis of HCC and/or end-stage liver disease (ESLD) is leading to increasing pressure in the waiting list for liver transplantation, with a consequent increasing rate of dropouts.

Beyond liver transplantation, several other therapeutic strategies are currently available for the management of early and intermediate-stage HCC, including hepatic resection, ablative therapies, and—more recently—systemic and immune-based treatments [2]. Hepatic resection remains the first-line curative option in patients with preserved liver function and resectable disease, while percutaneous ablative techniques such as radiofrequency or microwave ablation offer effective local control for small tumors, particularly in patients who are not ideal surgical candidates [3]. Systemic and immunotherapy regimens have progressively expanded the therapeutic armamentarium for intermediate and advanced stages, improving survival and allowing downstaging in selected cases [4].

Within this evolving framework, laparoscopic liver resection has emerged not only as a definitive curative treatment in appropriately selected patients but also as a strategic step within a “salvage transplantation strategy”, stabilizing oncologic disease while preserving the possibility of subsequent listing. LLR is considered safe and effective in selected patients, and its indications have expanded even to selected patients with portal hypertension and Child–Pugh B cirrhosis, provided careful patient selection and experienced surgical teams [5,6,7]. Such a treatment sequence aligns with contemporary models proposing a hierarchical integration of available therapies according to tumor stage, liver function, and transplant feasibility. This approach is particularly relevant in regions—such as southern Italy—where donor scarcity extends waiting times and increases the risk of dropout due to tumor progression or hepatic decompensation [8,9,10,11,12]. In this context, this study aimed to analyze the impact of laparoscopic liver resection on access to the waiting list at a single regional center for liver transplantation in southern Italy, a region with a donor shortage.

## 2. Materials and Methods

### 2.1. Study Design and Population

This was a retrospective single-center study including all consecutive patients evaluated and/or treated for ESLD and HCC at our regional liver transplant center between January 2015 and February 2023.

Patients were identified retrospectively from a prospectively maintained institutional database. The study was conducted in accordance with the ethical standards of the Declaration of Helsinki and the Declaration of Istanbul. According to local Institutional Review Board policy, specific ethical approval was not required for retrospective observational studies.

### 2.2. Inclusion and Exclusion Criteria

Inclusion criteria were: (1) patients with end-stage liver disease listed for liver transplantation between 2016 and 2023; (2) patients with a radiological or histological diagnosis of HCC who were considered suitable for surgical treatment (liver resection, thermal ablation, or liver transplantation) within the same period.

Patients who underwent living donor liver transplantation, combined liver transplantation, or pediatric transplantation were excluded.

#### Surgical Eligibility Criteria

Criteria for resectability included the absence of extrahepatic disease and of prohibitive comorbidities for surgery. The presence of portal hypertension was not considered an absolute contraindication unless associated with ascites or a history of variceal bleeding. Patients beyond the Milan criteria were considered for transplantation after individualized multidisciplinary evaluation.

### 2.3. Study Groups

Patients with potentially surgically treatable HCC were classified according to their eligibility for liver transplantation and the role of the initial treatment within the overall therapeutic options. This grouping reflected real-world clinical pathways in a region with a significant donor shortage, where treatment decisions must balance oncologic control and transplant eligibility. Given that both surgical resection and microwave thermal ablation (MWTA) are routinely used in our center as part of the therapeutic strategy for HCC, both as a bridging or downstaging therapy in transplant-eligible patients and as a definitive locoregional treatment in those who are not candidates for transplantation, MWTA-treated patients were incorporated into the study groups.

Patients considered for hepatic resection were divided into two predefined groups:

- Group A—Resectable but non-transplantable patients:

Patients ineligible for liver transplantation due to age >70 years, tumor burden beyond Milan criteria, or comorbidities contraindicating transplant. For these patients, resection represented the only potentially curative option.

- Group B—Resectable and transplantable patients:

Patients <70 years old, with HCC within Milan criteria, preserved performance status, and without medical contraindications to liver transplantation. Both Child–Pugh A and B patients were considered suitable for resection or trans-plantation. Portal hypertension, in the absence of ascites or variceal rupture, did not preclude surgery or transplant eligibility. In these cases, resection could serve either as a definitive treatment or as part of a staged pathway toward salvage transplantation.

#### Microwave Thermal Ablation Cohort

Similarly, patients undergoing microwave thermal ablation (MWTA) were divided into two groups based on their transplant candidacy:

- Bridge/Downstaging MWTA (Group C—transplantable–thermoablated):

Patients eligible for liver transplantation who received MWTA to maintain transplant candidacy (bridge therapy) or to reduce tumor burden with the objective of achieving transplant eligibility (downstaging).

- Non-transplantable MWTA (Group D—thermoablated only):

Patients not eligible for transplantation due to age, comorbidities, or tumor characteristics for whom MWTA was used as a definitive locoregional treatment.

A third group consisted of “transplantable-only” patients, included individuals with ESLD and patients with HCC directly listed for liver transplantation.

By distinguishing resection and MWTA cohorts according to transplantability, this framework allowed meaningful comparison of outcomes across clinically distinct pathways—curative-only versus curative-plus-transplant—and clarified the potential role of laparoscopic resection in mitigating waiting-list pressure in regions characterized by donor scarcity.

### 2.4. Data Collection

For each patient, the following variables were collected: demographic data, primary disease etiology, type and date of first-line treatment, liver function parameters, recurrence of HCC (presence, date, site, and treatment of recurrence), surgical approach (laparoscopic or open), type of resection (anatomical or wedge); for transplanted patients data regarding donor and graft characteristics (donor age, use of reconditioned grafts and perfusion technique, ischemia time); histopathology data were also collected (tumor grading, vascular invasion, Edmondson–Steiner grade, pathological tumor node metastasis—pTNM-stage).

### 2.5. Postoperative Management and Follow-Up

All transplanted patients received an internal immunosuppression protocol consisting of anti-IL-2 induction followed by tacrolimus maintenance therapy. Post-transplant follow-up included daily transabdominal ultrasound during the first postoperative week and periodic laboratory and imaging evaluations, progressively spaced to every six months by year three. Patients treated with hepatic resection for HCC underwent an oncologic follow-up consisting of abdominal CT and/or MRI, physical examination, and alpha-fetoprotein measurement every 3 months during the first year and annually thereafter.

### 2.6. Statistical Analysis

Categorical variables were summarized as counts and percentages, and continuous variables as medians with interquartile ranges, as appropriate. Group comparisons were performed using the Chi-square or Fisher’s exact test for categorical variables and the Mann–Whitney U or Kruskal–Wallis tests for continuous variables.

A linear correlation analysis was conducted to explore the relationship between the number of laparoscopic liver resections (LLR) for HCC and waiting list dynamics, including: patients listed at the beginning of each year, new annual enrolments, the Intention-to-Treat (ITT) population (patients listed at the beginning of the year plus new enrolments), patients remaining on the list at year-end, and the average waiting time for transplantation.

An ecological, year-by-year analysis was conducted to explore the association between LLR for HCC and the annual failure rate within the intention-to-treat (ITT) population, defined as all patients potentially eligible for liver transplantation. For each calendar year (2015–2016), the proportion of LLR among all hepatic resections was calculated, along with the yearly ITT failure rate, defined as the proportion of patients who exited the waiting list due to dropout or death. The strength and direction of the association between annual LLR rates and ITT failure rates were evaluated using Spearman’s rank correlation coefficient (ρ), given the limited sample size and non-normal data distribution.

A competing-risk analysis (Fine–Gray hazard model) was applied to estimate the cumulative incidence of dropout/death and curative treatment (liver transplantation, hepatic resection, or thermal ablation) as competing events among listed patients. Subdistribution hazard ratios (SHR) and 95% confidence intervals (CI) were reported.

All analyses adhered to the STROBE guidelines for observational studies [13]. Statistical significance was set at *p* < 0.05. Analyses were performed using Stata version 17.0 (StataCorp, College Station, TX, USA).

## 3. Results

A total of 884 patients were included in the study, enrolled between 2015 and 2023. The majority were male (73.8%), with a median age of 60 years (interquartile range, IQR, 54–70 years). Of these, 568 patients (64.3%) were classified as transplantable, 147 (16.6%) as transplantable–resected, 118 (13.4%) as resectable, 30 (3.4%) as transplantable–thermoablated, and 21 (2.4%) as thermoablated only (Table 1).

Among the 568 transplantable patients included in the study, 46.9% had HCC and 53.1% were listed for transplantation due to end-stage liver disease without HCC.

Regarding liver function, 43.0% of patients were classified as Child–Pugh A, 22.9% as B, and 34.1% as C, with significant differences among treatment groups (*p* < 0.001).

### 3.1. HCC Patients

Among the 881 patients included in the study, 581 (65.9%) were diagnosed with HCC. Of these, 118 (20.3%) underwent hepatic resection (group A), 147 (25.3%) were classified as transplantable–resected (group B), 21 (3.6%) underwent thermal ablation (group C), and 30 (5.2%) underwent MWTA as a bridge option to liver transplantation (group D). The remaining 265 patients (45.6%) with HCC were directly listed for liver transplantation. Median tumor size ranged from 12 mm (IQR 2.7–30) in Group B to 34 mm (IQR 20–54) in Group A patients. The majority of patients were within Milan criteria—54% in Group A, 77% in Group B patients, 85% in Group C treated with MWTA (Table 2).

### 3.2. Recurrence Patterns and Predictors

Among the surgically treatable HCC cohort (*n* = 316), 99 patients (31.3%) developed recurrence: 29 patients (25.22%) in the resectable cohort (group A), 48 patients (35.82%) in the resectable–transplantable cohort (group B), 7 patients (36.84%) in the termoablated cohort (group C) and 15 (53.57%) in the MWTA-transplantable cohort (group D). The multivariable logistic regression restricted to patients who recurred after hepatic resection (group A and B, *n* = 265) showed that belonging to the transplantable–resected group was independently associated with a lower risk of recurrence (OR = 0.05; 95% CI 0.003–0.81; *p* = 0.035). No significant associations were found for Child–Pugh class, Milan criteria, alpha-fetoprotein level, or R0/R1 status. Edmondson–Steiner grade showed a nonsignificant trend toward higher recurrence risk (OR = 2.45; 95% CI 0.93–6.48; *p* = 0.071). In the multivariable logistic regression analysis, which included all patients of the entire cohort who developed recurrence (*n* = 115), the only independent predictor of recurrence was belonging to the transplantable–thermoablated group (OR = 4.85, 95% CI 1.34–17.64, *p* = 0.016), as in Table 3. Other clinical and tumor-related variables, including Child–Pugh class, AFP, and Milan criteria status, were not found to be independently associated with recurrence.

Among patients of the entire cohort who developed recurrence (*n* = 115), the liver was by far the most frequent site of relapse (83.5%), followed by combined liver and lung recurrence (6.1%), isolated lung metastases (1.7%), and rarer extrahepatic sites such as bone (each < 1%).

Of the 93 patients with available treatment data, recurrence was managed mainly with loco-regional therapies (39.8%), followed by systemic or palliative treatment (21.5%), combined or sequential approaches (21.5%), and surgical re-intervention (15.1%).

There were no significant differences in the distribution of recurrence treatment among the primary therapeutic groups (Pearson χ^2^ = 16.2, *p* = 0.44).

Table 3. Multivariable logistic regression for predictors of recurrence in HCC patients after LT. HCC= hepatocellular carcinoma; LT= liver transplantation; LR= liver resection; MWTA= Microwave thermal ablation.

### 3.3. Trends of LLR and Dropout Rate

The proportion of laparoscopic liver resections increased steadily over the study period, rising from 48.6% in 2015 to 70.5% in 2023. This upward trend indicates a progressive adoption of minimally invasive techniques in our center, while the failure rate within the ITT ranged from 0% to 19.8%.

A strong inverse correlation was observed between the proportion of LLR and the failure rate (ρ = −0.78, *p* = 0.008), indicating that the growing adoption of laparoscopic liver resection was associated with a reduced number of drop out from the waiting list in a region characterized by limited organ availability.

In the competing-risk model (Fine–Gray regression) considering dropout, death, refusal, and non-eligibility as failures events and transplant, resection, or ablation as competing events, HCC patients showed a significantly lower sub-distribution hazard for failure compared to non-HCC patients (SHR = 0.27, 95% CI: 0.18–0.42, *p* < 0.001) (Table 4). The cumulative incidence function confirmed this finding: the probability of dropout/death over time was markedly higher in non-HCC patients, whereas HCC patients had a higher probability of reaching a competing event (LT/LR/MWTA). These results are consistent with the ecological trend observed in previous analyses, indicating that HCC patients maintain better access to transplantation and lower waiting-list mortality compared to non-HCC cases. In a complementary Fine–Gray model considering LT, LR, or MWTA as the goal event and dropout, death, non-eligibility as the competing event, HCC patients showed a significantly higher subdistribution hazard of achieving the goal event (SHR = 1.65; 95% CI 1.40–1.94; *p* < 0.001) (Table 4). This reinforces the finding from the failure-oriented model: HCC status is associated with both lower risk of negative exits and higher cumulative incidence of definitive therapy or transplantation, compared to non-HCC cases.

## 4. Discussion

Minimally invasive hepatic resection has become increasingly utilized for early stage HCC, especially in patients with compensated cirrhosis, due to its favorable perioperative outcomes, including reduced blood loss, lower morbidity, and shorter hospital stays compared to open resection [14,15,16,17,18]. Although these perioperative advantages are well established, the literature has not conclusively demonstrated that increased use of laparoscopic liver resection has a direct impact on liver transplantation waiting list pressure or dropout rates. The management of HCC with resection versus transplantation is determined by tumor burden, liver function, and presence of portal hypertension, with transplantation reserved for patients who are not candidates for resection or have multifocal disease or decompensated cirrhosis [14,15]. Reduction in waitlist dropout is generally achieved through bridging or downstaging therapies (TACE, ablation), rather than surgical resection [19,20].

In our experience, the increase in the rate of LLR among patients with HCC is inversely related to a significant reduction in waiting list pressure for liver transplantation, with a consequent reduction in the dropout rate. This inverse relationship underlines the impact of surgical therapies for HCC, especially LLR, for reducing the dropout rate and the waiting list time for liver transplantation: patients with early HCC who undergo LLR can reduce pressure on the waiting list for patients with non-HCC ESLD and maintain the probability of obtaining access to liver transplantation [21]. In a context of organ shortage and marginal grafts the combination of laparoscopic liver resection of HCC patients within Milan criteria, with possible salvage liver transplantation in case of early recurrence, and use of machine perfusion to expand the available donor pool, represents two strategies for reducing dropout rate and waiting list time that is associated with increased mortality [9,21].

HCC recurrence remains a major concern after LT, with rates reported between 12–17% in large cohorts [22], particularly in patients beyond Milan criteria or with aggressive tumor biology (high AFP, microvascular invasion) [16]. Multiple factors influence recurrence risk, including tumor size and number, AFP levels, vascular invasion, and underlying liver disease etiology [23,24]. Importantly, in our analysis, performing resection in patients who were also LT-eligible did not increase recurrence risk. This supports the feasibility of a strategy in which LLR serves as an initial curative step without compromising long-term oncologic outcomes in appropriately selected patients.

Accurate patient selection remains fundamental. While early hepatic resection is appropriate for patients with preserved liver function and limited tumor burden, the optimal sequencing of resection and transplantation continues to be debated. Some authors propose pre-emptive LT before recurrence in high-risk patients [25], whereas the classical salvage LT approach could remain feasible in case of HCC recurrence [26]. Current AASLD guidance supports salvage transplantation for selected patients, enabling those who remain within Milan criteria at recurrence to bypass the usual observation period for MELD exception points [1]. Nevertheless, defining which patients truly benefit from pre-emptive versus salvage LT remains challenging, and further evidence is needed. In this setting, living donor transplantation and the use of machine perfusion technologies may further expand graft availability and strengthen LT-based strategies [25].

Although LLR provides clear perioperative benefits and equivalent oncologic outcomes to open resection [15], evidence remains limited regarding its direct effect on transplant waiting list pressure at the population level [27]. Therefore, integrating tumor biology, radiologic response to locoregional therapies, and biomarkers into preoperative evaluation is essential for determining which patients may derive the greatest benefit from LLR versus upfront LT [27]. Moreover, given that early recurrence after LT (within 1–2 years) is associated with worse survival, individualized surveillance and management strategies remain critical [28].

In our single center experience, we evidenced that HCC patients showed a 73% lower risk of dropout/death compared to non-HCC. Particularly, HCC patients had a 65% higher subdistribution hazard of achieving LT or curative treatment compared to non-HCC. These findings align with existing evidence showing that HCC candidates—because of MELD exception points—experience lower dropout rates and higher transplant rates, even in regions with limited deceased-donor availability [1,28,29]. Although post-transplant survival for HCC remains slightly lower than for non-HCC recipients, mainly due to tumor recurrence, overall outcomes remain favorable, with 5-year survival of 65–80% [30]. At the population level, however, transplantation alone has a modest impact on overall HCC mortality, reinforcing the need for integrated approaches combining early detection, prevention, and optimized treatment sequencing [31,32].

Our experience suggests an inverse correlation between the increasing adoption of LLR and the rate of waiting list dropout, supporting its role as a bridge or alternative to transplantation within well-defined clinical criteria. However, current evidence remains insufficient to confirm a direct causal relationship between the broader use of LLR and reduced waitlist mortality at the population level. Careful patient selection, multidisciplinary evaluation, and integration of biological and radiological markers remain crucial in guiding individualized therapeutic strategies. Concurrently, advances such as machine perfusion and living donor transplantation offer promising avenues to expand the donor pool and complement surgical strategies aimed at reducing waitlist times and improving overall survival [33].

## 5. Conclusions

Minimally invasive LR has evolved into a safe and effective therapeutic option for selected patients with early-stage HCC and preserved liver function. Beyond its perioperative advantages, LLR contributes to optimizing the use of limited donor organs by offering a curative-intent alternative to transplantation in eligible candidates. In donor-scarce regions—where the transplant waiting list is populated by both patients with HCC and those with ESLD—this approach may indirectly alleviate waiting-list pressure and reduce dropout risk for the overall transplant population. Taken together, our findings support a dynamic and personalized management pathway in which minimally invasive resection, timely transplantation, and innovative graft-preservation techniques contribute synergistically to improving outcomes for patients with HCC within the complex context of ongoing organ scarcity.

## Figures and Tables

**Table 1:** Baseline characteristics of HCC patients—according to treatment group—and patients listed for liver transplantation.

	Surgically Treatable HCC Cohort (316, 35.7%)				Transplant Cohort (568, 64.3%)	*p* Value
	Group A	Group B	Group C	Group D		
Variable	Resectable (118, 13.4%)	Transplantable–Resectable (147, 16.6%)	MWTA Option (21, 2.4%)	Transplantable–MWTA Option (30, 3.4%)		
Age (years), median [IQR]	73.3 [68–78]	62.1 [57–67]	74.7 [72–77]	60.9 [57–66]	54.7 [49–61]	<0.001
BMI (kg/m^2^), median [IQR]	27.1 [25.0–29.2]	28.0 [25.3–31.1]	26.4 [23.9–29.0]	28.8 [25.6–31.3]	26.5 [23.1–29.5]	0.022
Sodium (mmol/L), median [IQR]	139.4 [138–141]	139.9 [138–142]	138.8 [137–141]	139.6 [138–142]	138.1 [136–141]	<0.001
Bilirubin (mg/dL), median [IQR]	0.57 [0.35–0.84]	0.68 [0.36–1.94]	0.84 [0.64–1.57]	1.05 [0.73–5.12]	2.08 [0.38–5.12]	<0.001
Creatinine (mg/dL), median [IQR]	0.94 [0.76–1.30]	0.94 [0.55–1.94]	0.92 [0.33–1.90]	0.97 [0.53–3.16]	1.01 [0.33–11.0]	0.014
INR, median [IQR]	1.07 [1.01–1.13]	1.07 [1.00–1.12]	1.07 [1.00–1.11]	1.15 [1.04–1.20]	1.43 [1.16–1.75]	<0.001
Platelet count (×10^9^/L), median [IQR]	189.5 [140–239]	176.1 [130–212]	169.5 [98–247]	115.3 [80–152]	106.3 [67–134]	<0.001
Alpha-fetoprotein (ng/mL), median [IQR]	5.8 [0.6–19.3]	12.9 [0.9–76.0]	4.0 [1.0–2999.0]	8.5 [1.0–6800.0]	5.6 [0.8–4568.0]	<0.001
Sex (male), *n* (%)	75 (63.6)	118 (80.3)	16 (76.2)	23 (76.7)	421 (74.1)	0.048
Child–Pugh A	99 (83.9)	112 (76.2)	11 (52.4)	22 (73.3)	136 (23.9)	<0.001
Child–Pugh B	1 (0.8)	3 (2.0)	3 (14.3)	2 (6.7)	193 (34.0)	
Child–Pugh C	18 (15.3)	32 (21.8)	7 (33.3)	6 (20.0)	239 (42.1)	
HCV-related	0 (0.0)	1 (0.7)	0 (0.0)	1 (3.3)	23 (4.1)	
HBV-related	10 (8.5)	15 (10.2)	1 (4.8)	2 (6.7)	80 (14.1)	
Alcohol-related	11 (9.3)	6 (4.1)	0 (0.0)	3 (10.0)	61 (10.8)	
NASH/Metabolic	0 (0.0)	1 (0.7)	0 (0.0)	1 (3.3)	15 (2.6)	
Autoimmune	6 (5.1)	17 (11.6)	3 (14.3)	3 (10.0)	47 (8.3)	
Cholestatic	18 (15.3)	20 (13.6)	3 (14.3)	8 (26.7)	84 (14.8)	
Other/Rare	61 (51.7)	66 (44.9)	13 (61.9)	9 (30.0)	217 (38.2)	0.011

**Table 2:** Characteristics of HCC patients in different groups according to primary treatment option.

Variable	Resectable—Group A (*n* = 118, 20.3%)	Transplantable–Resectable—Group B (*n* = 147, 25.3%)	MWTA—Group C (*n* = 21, 3.6%)	Transplantable–MWTA—Group D (*n* = 30, 5.2%)
Number of lesion, median [IQR]	1 [1–1]	1 [1–2]	1 [1–1]	1 [1–1]
Maximal diameter (mm), median [IQR]	34 [20–54]	12 [2.7–30]	22 [8.5–31]	15.5 [3.1–25]
Child–Pugh A, *n* (%)	99 (83.9)	112 (76.2)	11 (52.4)	22 (73.3)
Child–Pugh B, *n* (%)	1 (0.8)	3 (2.0)	3 (14.3)	2 (6.7)
Child–Pugh C, *n* (%)	18 (15.3)	32 (21.8)	7 (33.3)	6 (20.0)
Milan in, *n* (%)	51 (53.7)	113 (77.4)	11 (84.6)	28 (96.6)
Milano out, *n* (%)	30 (31.6)	24 (16.4)	1 (7.7)	1 (3.4)
No ESLD, *n* (%)	14 (14.7)	10 (6.2)	1 (7.7)	1 (0)
Surgical Approach				
Open	55 (46.6)	47 (32.0)	–	–
LLR	36 (30.5)	80 (54.4)	–	–
Hand-assisted option	27 (22.9)	20 (13.6)	–	–

**Table 3:** Multivariable logistic regression for predictors of recurrence in HCC patients after LT. HCC= hepatocellular carcinoma; LT= liver transplantation; LR= liver resection; MWTA= Microwave thermal ablation.

Variable	Odds Ratio (95% CI)	*p*-Value
Treatment group		
LT after LR	1.25 (0.53–2.93)	0.61
LT after MWTA	4.85 (1.34–17.64)	0.016
LT	0.12 (0.05–0.30)	<0.001
Child–Pugh class (B–C vs. A)	0.93 (0.63–1.38)	0.72
Milan criteria (out vs. in)	1.05 (0.82–1.34)	0.71
Alpha-fetoprotein (ng/mL)	1.00 (1.00–1.00)	0.39

**Table 4:** Fine–Gray competing risk analysis according to HCC status; HCC = hepatocellular carcinoma; LT = liver transplantation; LR = liver resection; MWTA = Microwave thermal ablation; HR = hazard ratio.

Event of Interest	Competing Event	Comparison (HCC vs. Non-HCC)	HR (95% CI)	*p*-Value	Interpretation
Failure (dropout, death, non-eligibility, refusal)	Goal (LT, LR, MWTA)	HCC vs. non-HCC	0.27 (0.18–0.42)	<0.001	HCC patients showed a 73% lower subdistribution hazard of dropout/death compared to non-HCC.
Goal (LT, LR, MWTA)	Failure (dropout, death, non-eligibility, refusal)	HCC vs. non-HCC	1.65 (1.40–1.94)	<0.001	HCC patients had a 65% higher subdistribution hazard of achieving transplantation or curative treatment compared to non-HCC.

## Data Availability

The data presented in this study are available on request from the corresponding author.

## Abbreviations

The following abbreviations are used in this manuscript:

AFP	Alpha-fetoprotein
ESLD	End-stage liver disease
HCC	Hepatocellular carcinoma
HOPE	Hypothermic oxygenated perfusion
LLR	Laparoscopic liver resection
LT	Liver transplantation
MWTA	Microwave thermal ablation
